# Molecular hydrogen as a treatment for ME/CFS: a mini-review of clinical evidence and mechanistic rationale

**DOI:** 10.3389/fmed.2026.1760210

**Published:** 2026-03-18

**Authors:** Fred Friedberg, Tyler W. LeBaron

**Affiliations:** 1 Department of Psychiatry and Behavioral Health, Stony Brook University Health Sciences Center, Stony Brook, NY, United States; 2 Department of Kinesiology and Outdoor Recreation, Southern Utah University, Cedar City, UT, United States; 3 Molecular Hydrogen Institute, Cedar City, UT, United States

**Keywords:** hydrogen-rich water, ME/CFS, mitochondrial dysfunction, molecular hydrogen, oxidative stress

## Abstract

Myalgic encephalomyelitis/chronic fatigue syndrome (ME/CFS) is a debilitating multisystem illness characterized by profound fatigue, post-exertional malaise, cognitive impairment, and autonomic dysfunction, yet it currently lacks FDA-approved treatments. Molecular hydrogen (H2), administered primarily as hydrogen-rich water (HRW), has emerged as a potential therapeutic candidate due to its selective antioxidant effects, anti-inflammatory activity, and support of mitochondrial and cellular homeostasis. These mechanisms align with several biological abnormalities implicated in ME/CFS, including oxidative stress, chronic inflammation, and impaired energy metabolism. This narrative mini-review summarizes mechanistic evidence relevant to ME/CFS and evaluates three developmental clinical studies of HRW in this population. Although early trials are small and methodologically limited, moderate-dose HRW consumed over extended durations has demonstrated feasibility and preliminary benefits in reducing fatigue and improving physical function, with generally mild side effects. Overlapping findings in Long COVID further suggest potential applicability across related post-viral fatigue conditions. Key limitations include small sample sizes, reliance on self-report outcomes, and the absence of objective biomarkers. Future research should prioritize larger, rigorously controlled trials incorporating remote biometric and biochemical assessments to clarify mechanisms of action and identify responsive subgroups. Overall, molecular hydrogen represents a promising, low-burden adjunctive therapy warranting further investigation in ME/CFS.

## 1 Introduction

Myalgic encephalomyelitis/chronic fatigue syndrome (ME/CFS) is a complex and often disabling illness characterized by the cardinal features of activity reduction or impairment, post-exertional malaise, unrefreshing sleep, and either cognitive impairment or orthostatic intolerance (1). Affecting millions worldwide, ME/CFS poses a substantial burden on quality of life, yet it remains poorly understood and lacks any FDA-approved treatments. Conventional therapies are limited to symptomatic management and supportive care (2).

The underlying pathophysiology of ME/CFS is heterogeneous and multifactorial. Among the most consistently implicated biological abnormalities are mitochondrial dysfunction, oxidative stress, chronic inflammation, and autonomic nervous system dysregulation (3–5). These processes are believed to contribute to impaired energy metabolism, neurocognitive symptoms, and heightened sensitivity to physical and psychological stress (5). Biomarker development has been limited, and the absence of objective assessment tools continues to hinder both diagnosis and evidence-informed treatment evaluation (6).

In light of these challenges, there is growing interest in exploring therapies that target multiple overlapping pathological domains. Molecular hydrogen (H_2_ gas) has emerged as a potential candidate due to its selective antioxidant properties, anti-inflammatory effects, and support of mitochondrial and cellular homeostasis (7). Hydrogen gas can be administered safely with hydrogen-rich water (HRW), which is generated by dissolving specialized magnesium-based tablets in water, producing therapeutic concentrations of dissolved H2 gas. Hydrogen gas has been granted “Generally Recognized as Safe” (GRAS) status by the FDA for use in beverages.

Recent pilot studies using HRW in ME/CFS have yielded encouraging findings, including improvements in fatigue and physical function, with a favorable safety profile (8, 9). Given these early observations and the mechanistic plausibility of H2 therapy, this narrative mini-review summarizes the rationale for using molecular hydrogen in ME/CFS and critically examines available clinical studies in this population.

## 2 Mechanistic rationale for hydrogen therapy in ME/CFS

Molecular hydrogen (H2) is a small, neutral molecule that can rapidly diffuse across cellular membranes, including the blood-brain barrier (10). When dissolved in water and ingested as hydrogen-rich water (HRW), H2 distributes systemically and reaches target tissues where it can exert cytoprotective effects (11). It has been extensively studied in both preclinical and clinical contexts, demonstrating safety and putative efficacy in a wide range of disease models, including those characterized by oxidative stress and inflammation (12).

A central mechanism of H2 is its selective antioxidant action. Unlike conventional antioxidants, H2 does not indiscriminately scavenge all reactive oxygen species (ROS). Instead, it selectively reduces the most cytotoxic ROS, namely hydroxyl radicals (OH) and peroxynitrite (ONOO^–^), while sparing signaling molecules such as superoxide and hydrogen peroxide (13). This allows H2 to mitigate oxidative damage without disrupting essential redox-dependent signaling pathways.

Additionally, H2 modulates inflammatory signaling. It has been shown to downregulate key pro-inflammatory cytokines including interleukin-6 (IL-6) and tumor necrosis factor-alpha (TNF-α), possibly via inhibition of the nuclear factor-kappa B (NF-κB) pathway (14). Given the elevated levels of these cytokines observed in subsets of ME/CFS patients (15), this anti-inflammatory effect may be particularly relevant.

Another important mechanism involves mitochondrial function which has been implicated in ME/CFS and may underlie the hallmark symptoms of fatigue and post-exertional malaise (16). H2 has been reported to stabilize mitochondrial membrane potential, reduce mitochondrial-derived ROS, and enhance endogenous antioxidant defenses by activating the Nrf2 pathway (17). These effects contribute to improved mitochondrial efficiency and cellular energy metabolism. Recent work identified the Rieske iron–sulfur protein (RISP) in complex III as a primary molecular target of H2, showing that H2 triggers LONP1-mediated degradation of RISP, transiently suppressing complex III activity and ATP production (18). This initial inhibition then induces the mitochondrial unfolded protein response (UPRmt) and a compensatory upregulation of respiratory chain components, consistent with a mitohormetic mechanism by which H2 ultimately enhances mitochondrial resilience and homeostasis (18).

In addition, H2 may exert neuroprotective effects by reducing neuroinflammation and oxidative injury in the central nervous system (19). Given the frequent occurrence of neurocognitive symptoms in ME/CFS, such as memory deficits and brain fog, this mechanism is of particular interest. H2 may also promote mild hormetic responses, which are adaptive cellular stress responses that increase resilience to oxidative and inflammatory insults (20).

Finally, H2 may influence autonomic nervous system function in an illness such as ME/CFS which has been associated with reduced heart rate variability (HRV), reflecting impaired parasympathetic tone (21). In an initial 4-weeks double-blinded, placebo-controlled study of HRW on the quality of life of healthy adult volunteers (22), the HRV findings suggested that both sympathetic and vagal mechanisms were significantly affected in the HRW group but not in the control condition.

## 3 Clinical studies of HRW in ME/CFS

To date, three clinical studies have investigated the use of hydrogen-rich water (HRW) in individuals with ME/CFS, all led by the same investigative team. These pilot trials varied in dosing, treatment duration, and methodology but together provide a preliminary foundation for future research. The initial study (23) was a 4-weeks pilot randomized controlled trial (RCT) which scheduled a high daily dose of HRW. This involved consuming five glasses daily of HRW (≈12 mg H_2_/day) by dissolving one tablet in 250 mL of water (10 mg/L) in each glass. Subjects filled a glass with 250 mL of tap water at room temperature, added an assigned tablet, allowed the tablet to dissolve for 90 s, and then drank the entire glass of HRW all at once, as hydrogen gas quickly dissipates. The study reported no significant symptom or functional benefits, nor changes in assessed biological markers of heart rate variability (HRV) and salivary uric acid. Approximately half of the participants experienced temporary moderate-to-severe adverse effects, including headaches and gastrointestinal discomfort. Although minimal adverse effects have been reported in H_2_ inhalation and HRW trials in the medical literature for chronic conditions such as metabolic syndrome and diabetes (24), the apparent heightened medication sensitivity often reported in the ME/CFS population may be an exception. Alternatively, it is plausible that the non-standard higher H_2_ dosage itself (>12 mg/day), rather than a specific illness sensitivity in this HRW trial triggered the unexpected adverse effects. In addition, the 30-days treatment duration may have been too short to adequately test the intervention.

Given the above considerations, a remotely conducted second pilot randomized trial (8) utilized a more typical standard dose (≈>7.5 mg) of HRW consisting of 1 tablet in 250 mL water (>10 mg/L three times per day) for an extended duration of 8 weeks. The trial also randomized participants to a comparison condition of heart rhythm biofeedback. In this remotely administered pilot trial, the HRW condition yielded small but statistically significant improvements in fatigue and self-report physical function. Side effects were generally mild, consisting of gastric symptoms and headaches, and were less frequent than in the initial trial. The biofeedback group did not show any significant benefit.

Given the modestly improved clinical outcomes in the 8-weeks trial (8), a recently completed study (9) extended the treatment period to 16 weeks of home-based consumption of hydrogen water. Participants were randomized to either a standard dose condition of HRW for the full 16 weeks or a dose escalation condition (standard dose for 8 weeks followed by an elevated dose for 8 weeks). Both dosing strategies resulted in statistically significant and clinically relevant improvements in fatigue and physical function (23). However, only the standard-dose group showed additional benefits for depression and anxiety scores. No significant changes were found in salivary uric acid levels, which had been hypothesized as a potential biomarker. For the full cohort, attrition was moderate (26%) due largely to ongoing illness challenges as reported by subjects. The lack of significant differences between the two dosing conditions suggests that a standard dose may be sufficient to achieve clinically meaningful outcomes. These three developmental studies collectively support the feasibility and preliminary efficacy of HRW in ME/CFS, particularly at moderate doses over longer treatment durations.

## 4 Relevance to long COVID and overlapping mechanisms

The emerging condition known as Long COVID, or post-COVID condition, shares significant clinical overlap with ME/CFS, including symptoms such as fatigue, dyspnea, cognitive impairment, and post-exertional malaise (24). These symptoms often persist well beyond viral clearance and may be driven by sustained oxidative stress, inflammation, and metabolic dysfunction (25). Given these similarities, findings from studies involving HRW in Long COVID may have translational relevance to ME/CFS. For example, Tan et al. (26) conducted a 14-days single-blind placebo-controlled pilot RCT of HRW in individuals with Long COVID. The intervention group reported significantly reduced fatigue on the Fatigue Severity Scale (*p* < 0.05) and greater distance in the 6-min walk test (*p* < 0.001), although no significant changes were observed in dyspnea or depression.

Apparent overlap in both symptomatology and treatment response between Long COVID and ME/CFS suggest that these conditions may share underlying pathophysiological mechanisms, including elevated oxidative stress (24, 26, 27). The modest but meaningful clinical responses to HRW observed in both populations support further investigation of H2 as a potentially unifying therapeutic approach.

## 5 Limitations and challenges in HRW research for ME/CFS

Despite promising early findings, several methodological and practical limitations constrain the current body of evidence evaluating HRW in ME/CFS. First, the sample sizes in all published trials have been small, limiting statistical power and generalizability. Only one study (8) employed a placebo-controlled design, while the others used open-label or active comparator formats. Second, outcome assessments relied heavily on self-report measures of fatigue and self-reported physical function. Although these are clinically appropriate assessments commonly used in ME/CFS research, they are susceptible to bias, particularly inaccurate recall in multi-month trials with limited assessments (28). The use of frequently scheduled digitized assessments of self-report fatigue, physical function and other quality of life measures is likely to lessen recall bias, given shorter recall intervals as well as data entry convenience, particularly in home-based trials (28). To reduce recall bias in our 3 remotely administered HRW trials, weekly digital diary assessments of symptoms were scheduled in addition to pre- and post-trial assessments using validated questionnaires.

Other limitations of the ME/CFS hydrogen water trials include the absence of objective biomarkers which remain an ongoing scientific challenge. Furthermore, these remotely conducted trials restrict the ability to gather objective physiological data, although such studies have unique advantages, e.g., improved accessibility particularly for homebound patients who represent a substantial proportion (about 25%) of the ME/CFS population (29). Finally, variability in dosing protocol and treatment duration across trials, make direct comparisons difficult. The absence of clear guidelines for optimal H2 dosage and duration in ME/CFS leaves a gap in protocol standardization (30). Taken together, these limitations underscore the need for larger, well-controlled trials incorporating both subjective and objective outcomes, along with standardized treatment protocols and rigorous blinding procedures.

As future trials incorporate objective biomarker strategies, these tools may also enable clearer evaluation of proposed mechanisms in hydrogen water treatment. The growing body of evidence on molecular hydrogen’s antioxidant and anti-inflammatory actions (31) underscores the relevance of measuring oxidative stress and related metabolic pathways in ME/CFS. Integrating such biomarker assessments into remote HRW trials would help to determine whether the physiological effects attributed to hydrogen are reflected in ME/CFS patients and clarify the mechanisms underlying the preliminary clinical benefits observed to date.

## 6 Future directions

What is now needed are well-powered, randomized controlled trials incorporating biomarker assessments to elucidate likely mechanisms such as oxidative stress and systemic inflammation. For example, the use of convenient, low-cost blood collection devices for remote biomarker assessment would represent an important advance for an illness currently lacking objective measures of diagnosis, disease severity, or clinical outcomes. To that end, the development of portable devices for biomarker assessment has recently produced new low burden technologies that allow individuals to self-collect capillary blood samples for metabolomics analyses. These portable devices (e.g., Tasso+ and TAP devices) may be utilized for at-home testing and/or therapeutic monitoring. Findings from a recent analysis of self-collected capillary blood samples from five healthy volunteers (30) indicated that self-use shoulder-microblade devices yielded sufficient sample volumes and results comparable to those obtained from laboratory-collected venous blood. These devices are largely painless, less expensive, and more accessible than phlebotomist-administered venipuncture, making them appropriate for self-administration. This recently developed technology represents a promising new direction to advance remotely delivered studies that can now incorporate objective data collection at key study milestones (e.g., treatment midpoint, termination, etc.), thereby facilitating biomarker discovery in complex conditions such as ME/CFS.

## 7 Conclusion

This brief review highlighted the therapeutic potential of molecular hydrogen in ME/CFS in initial trials. To reach limited-mobility patients, hydrogen water is a feasible, low burden intervention that is easily adapted to home-based administration. Initial studies suggest potential benefits in reducing fatigue and improving overall symptom burden, consistent with molecular hydrogen’s reported antioxidant and anti-inflammatory properties. While evidence remains preliminary, these findings provide a rationale for continued investigation of HRW as a safe and practical adjunctive treatment in ME/CFS, ideally through rigorously designed, biomarker-integrated clinical trials.
